# Learning to Sense for Coded Diffraction Imaging

**DOI:** 10.3390/s22249964

**Published:** 2022-12-17

**Authors:** Rakib Hyder, Zikui Cai, M. Salman Asif

**Affiliations:** Department of Electrical and Computer Engineering, University of California, Riverside, CA 92521, USA

**Keywords:** phase retrieval, coded diffraction imaging, learned sensors

## Abstract

In this paper, we present a framework to learn illumination patterns to improve the quality of signal recovery for coded diffraction imaging. We use an alternating minimization-based phase retrieval method with a fixed number of iterations as the iterative method. We represent the iterative phase retrieval method as an unrolled network with a fixed number of layers where each layer of the network corresponds to a single step of iteration, and we minimize the recovery error by optimizing over the illumination patterns. Since the number of iterations/layers is fixed, the recovery has a fixed computational cost. Extensive experimental results on a variety of datasets demonstrate that our proposed method significantly improves the quality of image reconstruction at a fixed computational cost with illumination patterns learned only using a small number of training images.

## 1. Introduction

Coded diffraction imaging is a specific instance of Fourier phase retrieval problems. Phase retrieval refers to a broad class of nonlinear inverse problems where we seek to recover a complex- (or real-) valued signal from its phase-less (or sign-less) measurements [1,2,3,4]. In practice, these problems often arise in coherent optical imaging where an image sensor records the intensity of the Fourier measurements of the object of interest. In coded diffraction imaging, the signal of interest is modulated by a sequence of known illumination patterns/masks before observing the Fourier intensity at the sensor [2,4]. Applications include X-ray crystallography [5,6], astronomy [7,8], microscopy [9,10,11,12], speech processing and acoustics [13,14], and quantum mechanics [15,16]. Similar to other signal recovery problems in various imaging and signal processing tasks [4,5,11,17,18], iterative methods are also used in coded diffraction imaging. In this paper, we present a framework to design the illumination patterns for better signal recovery for coded diffraction imaging using a fixed-cost iterative method in a data-driven manner.

Let us denote the signal of interest as $x\in R^{n}$ or $C^{n}$ that is modulated by *T* illumination patterns $D=\{d_{1},\ldots ,d_{T}\}$, where $d_{t}\in R^{n}$ or $C^{n}$. The amplitude of sensor measurements for $t^{\mathrm{th}}$ illumination pattern can be written as

(1) $$y_{t}=\left|F\left(d_{t}⊙x\right)\right|,$$

where F denotes the Fourier transform operator, and ⊙ denotes an element-wise product. We note that real sensor measurements are proportional to the intensity of the incoming signal (i.e., square of the Fourier transform). In practice, however, solving the inverse problem with (non-square) amplitude measurements provides better results [19,20]; therefore, we use the amplitude measurements throughout the paper.

To recover the signal *x* from the the observed measurements, we can solve the following optimization problem:

(2) $$\min_{x}\;\sum_{t=1}^{T}∥y_{t}-|F\left(d_{t}⊙x\right){|∥}_{2}^{2}.$$

In recent years, a number of iterative algorithms have been proposed for solving the problem in (2), which includes lifting-based convex methods, alternating minimization-based non-convex methods, and greedy methods [2,21,22,23,24].

**Figure 1:**
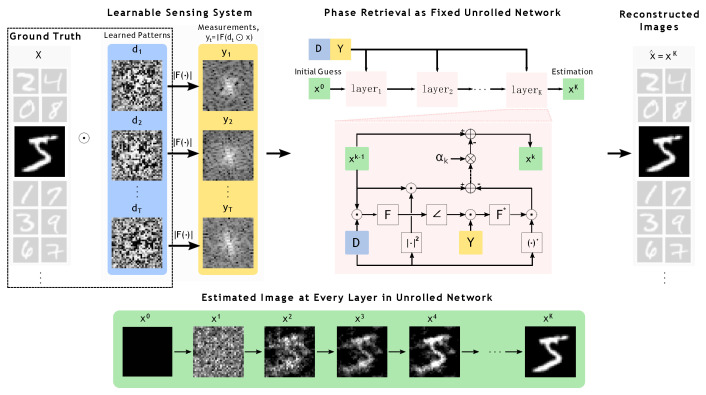
Pipeline of our proposed framework at inference time. Our framework mainly contains two components: (1) a learnable sensing system that updates the illumination patterns during training time, but at inference time the learned illumination patterns are fixed; (2) a fixed unrolled network that runs phase retrieval process to recover the original signal *x* form measurements *Y*. The number of layers in the network is fixed to *K*. Steps at every iteration are fixed and depicted as an unrolled network (details can be found in Algorithm 1).

Our goal is to learn a set of illumination patterns to optimize the recovery of an alternating minimization (AltMin) algorithm for solving the problem in (2). The AltMin method can be viewed as an unrolled gradient descent network, as shown in Figure 1, where we fix the steps at every iteration and the total number of iterations for AltMin. One forward pass through the unrolled network is equivalent to *K* iterations of the AltMin algorithm using given illumination patterns. We can increase or decrease the number of iterations for better accuracy or faster run-time. To keep the computational complexity of the recovery algorithm low, we keep the total number of iterations small (e.g., K=50). At the training stage, we optimize over the illumination patterns to minimize the error between the AltMin outputs after *K* iterations and the ground truth training images. At the test time, we solve the problem in (2) using *K* AltMin iterations with the learned illumination patterns (equivalent to one forward pass). We evaluated our method on different image datasets and compared against existing methods for coded diffraction imaging. We demonstrate that our proposed method of designing illumination patterns for a fixed-cost algorithm outperforms existing methods both in terms of accuracy and speed.

The main contributions of this paper are as follows.

- **Low cost inference**: We learn illumination patterns for coded diffraction imaging using the unrolled network formulation of a classical AltMin method. We show that with our learned illumination patterns, the unrolled AltMin method outperforms other computationally complex algorithms and provides superior image reconstruction within a much shorter time.
- **Learning from small dataset**: We use only a small number of training samples and can learn illumination patterns that are highly effective for image reconstruction. It is crucial for real-life applications because finding training samples can be challenging in practice.
- **Robust sensor design**: The patterns learned on a given dataset generalize to different datasets and provide robust reconstruction for shifted and flipped versions of the target samples. It does not drastically degrade under noisy measurements. Our learned illumination patterns can also help other algorithms achieve better performance even though they are not used for training.

## 2. Related Work

**Phase Retrieval and Coded Diffraction Patterns.** A Fourier phase retrieval problem arises in a number of imaging systems because standard image sensors can only record intensity of the observed measurements. This problem has been extensively studied over the last five decades in optics, signal processing, and optimization [3,4,5,25,26]. Coded diffraction imaging is a physically realistic setup in which we can first modulate the signal of interest and then collect the intensity measurements [18,27]. The modulation can be performed using a spatial light modulator or custom transparencies [10,11,28]. The recovery problems involve solving a phase retrieval problem; the presence of modulation patterns makes this a more tractable problem compared to classical Fourier phase retrieval [18].

The algorithms for solving phase retrieval problem can be broadly divided into non-convex and convex methods. Classical algorithms for phase retrieval rely on solving the underlying non-convex problem using alternating minimization. Amplitude flow [29,30], Wirtinger flow [31,32], and alternating minimization (AltMin) [22,23,33] are such methods that solve the non-convex problem. Convex methods usually lift the non-convex problem of signal recovery from quadratic measurements into a convex problem of low-rank matrix recovery from linear measurements. The PhaseLift algorithm [2] and its variations [18,21] can be considered under this class. Other algorithms, such as PhaseMax [34,35] and PhaseLin [36], use convex relaxation to solve the non-convex phase retrieval problem without lifting the problem to a higher dimension. We can also incorporate prior knowledge about the signal structure (e.g., sparsity, support, or positivity) in the recovery process constraints [22,29,32,37,38].

**Data-Driven Approaches for Phase Retrieval.** Recently, the idea of replacing the classical (hand-designed) signal priors with deep generative priors for solving inverse problems has been explored in different works [39,40]. Refs. [23,26,41,42,43,44] focused especially on solving phase retrieval problems with generative priors. Another growing trend is learning the solution of inverse problems (including phase retrieval) in an end-to-end manner, where deep networks are trained to learn a mapping from sensor measurements to the signal of interest using a large number of measurement-signal pairs. A few examples demonstrating the benefit of the data-driven approaches include robust phase retrieval [20], Fourier ptychographic microscopy [45], holographic image reconstruction [46], and correlography for non-line-of-sight imaging [47].

Although our method is partially driven by data, our goal is not to learn a signal prior or a mapping from measurements to signal. We use a very small dataset (consisting 32 or 128 images only) to learn the illumination patterns for a fixed recovery algorithm. Furthermore, the patterns we learn on one class of images provide good results on other types of images. Apart from the great flexibility and generalization, our method uses a fixed number of iterations of the well-defined AltMin routine, which is parameter-free during inference (except the step size) compared to end-to-end or generative prior-based approaches.

The approach we used for optimizing over the AltMin routine to learn illumination patterns is broadly known as unrolling networks. Iterative methods for solving the inverse problems, such as AltMin or other first-order methods, can be represented as unrolled networks. Every layer of such a network performs the same steps as a single iteration of the original method [48,49,50,51,52,53,54,55,56,57]. Some parameters of the iterative steps can be learned from data (e.g., step size, denoiser, or threshold parameters), but the basic structure and physical forward model are kept intact.

**Learn to Sense.** Data-driven deep learning methods have also been used to design the sensing system, especially in the context of compressive sensing and computational imaging [58,59,60,61,62,63]. The main objective in these methods is similar to ours, which is to find the sensor parameters to recover the best possible signal/image from the sensor measurements. The sensor parameters may involve selection of samples/frames, design of sampling waveforms, or illumination patterns as we discuss in this paper. In contrast to most of the existing methods that learn a deep network to solve the inverse problem, our method uses a predefined iterative method as an unrolled network, while learning the illumination patterns using a small number of training images. Unrolled networks for solving non-linear inverse problems have been used in [45,64]. Ref. [45] proposes learning sensors for Fourier ptychographic microscopy, whereas [64] designs sensing patterns for coded illumination imaging. One might find a similarity between [64] and our problem formulation. In principle, the sensor can be treated as the first layer of the network with some physical constraints on the parameters [64]. However, the method in [64] uses an unrolled network to learn the sensing parameters for a quantitative phase imaging problem under the “weak object approximation”. This approximation turns the original nonlinear problem into a linear inverse problem. This assumption is only applicable where the target objects have a small scatter term (e.g., biological samples in closely index-matched fluid). In our setup, we do not make any such assumptions on target object and solve the original nonlinear coded diffraction imaging problem. This potentially makes our algorithm suitable for more general applications than [64].

## 3. Proposed Method

Our proposed method for learning illumination patterns can be divided into two parts. The first (inner) part involves solving the phase retrieval problem with given coded diffraction patterns using AltMin as an unrolled network (see block diagram in Figure 1). The second part is updating the illumination patterns based on backpropagating the image reconstruction loss. These two parts provide optimized image reconstruction and illumination patterns. Pseudocodes for both parts are listed in Algorithms 1 and 2.
**Algorithm 1**solveCDP(Y,D) via alternating minimization using single-step gradient descent**Input:** Measurements $Y=\{y_{1},\ldots ,y_{t}\}$ and illumination patterns $D=\{d_{1},\ldots ,d_{T}\}$.**Initialization:** Zero initialization of estimate $x^{0}$.**for**k=1,2,…,K**do**                     ▹*K* iterations of AltMin      $p_{t}^{k-1}\leftarrow \mathrm{phase}(F\left(d_{t}⊙x^{k-1})\right)$ for all *t*.      $\nabla_{x}L_{x,p}=\frac{2}{T}\sum_{t=1}^{T}\left[\right|d_{t}{|}^{2}⊙x^{k-1}-d_{t}^{*}⊙F^{*}\left(p_{t}^{k-1}⊙y_{t}\right)]$      $x^{k}\leftarrow x^{k-1}-\alpha \nabla_{x}L_{x,p}$      Project $x^{k}$ onto feasible range.**end for****Output:** Estimated signal $x^{K}$.

**Algorithm 2** Learning illumination patterns
**Input:** Training set *X* with *N* images $X=\{x_{1},\ldots ,x_{N}\}$.**Initialize:** Initialize the optimization variables for *T* patterns as $\Theta =\{\theta_{1},\ldots ,\theta_{T}\}$ from a uniform distribution.**for**epoch=1,2,…,M**do**                            ▹*M* epochs      Generate illumination patterns $d_{t}=\mathrm{sigmoid}\left(\theta_{t}\right)$for all *t*      **for** n=1,2,…,N **do**                             ▹*N* samples            $Y_{n}=\{y_{1,n},\ldots ,y_{T,n}\;|\;y_{t,n}=|F\left(d_{t}⊙x_{n}\right)\left|\right\}$            $x_{n}^{K}\left(\Theta \right)\leftarrow \mathrm{solveCDP}$(*Y*_*n*_,*D*)      **end for**      $L_{\Theta }=\sum_{n=1}^{N}{∥x_{n}-x_{n}^{K}\left(\Theta \right)∥}_{2}^{2}$      $\Theta \leftarrow \Theta -\beta \nabla_{\Theta }L_{\Theta }$                       ▹ Update Θ with stepsize β
**end for**
**Output:** Learned illumination patterns $D=\{d_{1},\ldots ,d_{T}\;|\;d_{t}=\mathrm{sigmoid}\left(\theta_{t}\right)\}.$


We use *N* training images ($x_{1},\ldots ,x_{N}$) to learn *T* illumination patterns that provide the best reconstruction using a predefined (iterative) phase retrieval algorithm. Furthermore, to ensure that the illumination patterns are physically realizable, we constrain their values to be in the range [0,1]. We use a sigmoid function over unconstrained parameters $\Theta =\{\theta_{1},\ldots ,\theta_{T}\}$ to define the illumination patterns; that is, $d_{t}=\mathrm{sigmoid}\left(\theta_{t}\right)$ for all t=1,…,T.

**Phase retrieval with alternating minimization (AltMin).** Given measurements $Y=\{y_{1},\ldots ,y_{T}\}$ and illumination patterns $D=\{d_{1},\ldots ,d_{T}\}$, we seek to solve the CDP phase retrieval problem by minimizing the loss function defined in (2) as

(3) $$L_{x}=\frac{1}{2}\sum_{t=1}^{T}∥y_{t}-|F\left(d_{t}⊙x\right){|∥}_{2}^{2}.$$

Although the loss function in (3) is non-convex and non-smooth with respect to *x*, we can minimize it using the well-known alternating minimization (AltMin) with gradient descent [22,33]. In AltMin formulation, we define a new variable for the estimated phase of linear measurements as $p_{t}=\mathrm{phase}\left[F\left(d_{t}⊙x\right)\right]$ and reformulate the loss function in (3) into

(4) $$L_{x,p}=\frac{1}{2}\sum_{t=1}^{T}{∥p_{t}⊙y_{t}-F\left(d_{t}⊙x\right)∥}_{2}^{2}.$$

The gradient with respect to *x* can be computed as

(5) $$\nabla_{x}L_{x,p}=\sum_{t=1}^{T}{\left|d_{t}\right|}^{2}⊙x-d_{t}^{*}⊙F^{*}\left(p_{t}⊙y_{t}\right),$$

where $F^{*}$ denotes the inverse Fourier transform, and $d_{t}^{*}$ is the conjugate of pattern $d_{t}$. We can update the estimate at every iteration as

(6) $$x^{k}=x^{k-1}-\alpha_{k-1}\nabla_{x}L_{x,p},$$

where $\alpha_{k-1}$ denotes the step size. Another way is to directly solve for $x^{k}$ such that $\nabla_{x}L_{x,p}=0$. The closed-form solution is

(7) $$x^{k}=(\sum_{t=1}^{T}|d_{t}{{|}^{2})}^{-1}⊙\left[\sum_{t=1}^{T}d_{t}^{*}⊙F^{*}\left(p_{t}^{k-1}⊙y_{t}\right)\right].$$

We compared these two strategies and found that single-step gradient descent tends to work well in practice, and the closed-form solution does not show an advantage over the single-step gradient descent. In our implementation, we used the former strategy (Algorithm 1) and fixed a step size α for all iterations. The unrolled network has *K* layers that implement *K* iterations of the gradient descent, and the final estimate is denoted as $x^{K}$.

Choice of initialization is important, and our method can handle different types of initialization. Zero initialization, where every pixel of the initial guess of $x^{0}$ is 0, is the simplest and cost-free method. Many recent phase retrieval algorithms [30,31,33,35] use spectral initialization, which tries to find a good initial estimate. However, it requires computing the principal eigenvector of the following positive semidefinite matrix, $\sum_{t=1}^{T}\mathrm{diag}\left(d_{t}^{*}\right)F^{*}\mathrm{diag}(|y_{t}{|}^{2})F\mathrm{diag}\left(d_{t}\right)$. In our experiments, we observed that spectral initialization does not provide a significant improvement in terms of image reconstruction and that our algorithm can perform very well using the overhead-free zero initialization.

**Learning illumination patterns.** To learn a set of illumination patterns that provide the best reconstruction with the predefined iterative method (or the unrolled network), we seek to minimize the difference between the original training images and their estimates. In this regard, we minimize the following quadratic loss function with respect to Θ:

(8) $$L_{\Theta }=\frac{1}{2}\sum_{n=1}^{N}{∥x_{n}-x_{n}^{K}\left(\Theta \right)∥}_{2}^{2},$$

where $x_{n}^{K}\left(\Theta \right)$ denotes the solveCDP estimate of *n*th training image for the given values of Θ. Note that for given real values of $\Theta =\{\theta_{1},\ldots ,\theta_{T}\}$, we can define illumination patterns as $d_{t}=\sigma \left(\theta_{t}\right)$, where σ(·) is the *sigmoid* function. We can define sensor measurements for $x_{n}$ as $y_{t,n}=\left|F\left(d_{t}⊙x_{n}\right)\right|=p_{t,n}^{*}⊙F\left(d_{t}⊙x_{n}\right)$ for t=1,…,T and n=1,…,N, where $p_{t,n}=\mathrm{phase}\left[F\left(d_{t}⊙x_{n}\right)\right]$ is the phase of the original complex-valued signal.

We can use the recursive expression of the signal estimate in (6) and the gradient in (5) to represent the estimate of $x_{n}$ at iteration/layer *k* with the given values of Θ as

(9) $$x_{n}^{k}\left(\Theta \right)=(1-\alpha \sum_{t=1}^{T}|d_{t}{|}^{2})x_{n}^{k-1}\left(\Theta \right)+\alpha \sum_{t=1}^{T}d_{t}^{*}⊙F^{*}\left(p_{t,n}^{k-1}⊙y_{t,n}\right),$$

where $p_{t,n}^{k}=\mathrm{phase}\left[F\left(d_{t}⊙x_{n}^{k}\left(\Theta \right)\right)\right]$. We can compute the gradient of the loss function in (8) with respect to any $\theta_{t}$ in a recursive manner as follows.

(10) $$\begin{array}{c} \nabla_{\theta_{t}}L_{\Theta }=\sum_{n=1}^{N}J_{\theta_{t}}\left(x_{n}^{K}\left(\Theta \right)\right)\left[x_{n}^{K}\left(\Theta \right)-x_{n}\right], \end{array}$$

where $J_{\theta_{t}}\left(x_{n}^{K}\left(\Theta \right)\right)$ denotes the Jacobian matrix of the signal estimate with respect to $\theta_{\tau }$. We can now write the product of the Jacobian matrix with a vector *u* as

(11) $$\begin{array}{cc} \; & J_{\theta_{\tau }}\left(x_{n}^{K}\left(\Theta \right)\right)\left[u\right]=J_{\theta_{\tau }}\left(x_{n}^{K-1}\left(\Theta \right)\right)[(1-\alpha \sum_{t=1}^{T}|d_{t}{|}^{2})⊙u]\\ \; & -2\alpha |d_{\tau }{|}^{2}⊙\left(1-d_{\tau }\right)⊙x_{n}^{K-1*}\left(\Theta \right)⊙u\\ \; & +\alpha d_{\tau }⊙\left(1-d_{\tau }\right)⊙F^{*}\left(p_{\tau ,n}^{K}⊙y_{\tau ,n}\right)⊙u\\ \; & +\alpha d_{\tau }⊙\left(1-d_{\tau }\right)⊙x_{n}⊙F^{*}\left(p_{\tau ,n}⊙p_{\tau ,n}^{K*}⊙F\left(d_{\tau }⊙u\right)\right), \end{array}$$

where $J_{\theta_{\tau }}\left(x_{n}^{0}\right)=0$ for all n,τ. Here, we assume initial estimate $x_{n}^{0}=0$ and $\alpha_{k}=\alpha$ for k=1,…,K. We also assume that the phase of the measurements or the signal estimates do not change with small changes in Θ. The overall gradient of the reconstruction loss with respect to the parameters Θ can be computed in a recursive manner (backpropagation ) using element-wise products and forward/inverse Fourier transform operations at every iteration/layer.

We can use gradient descent to find the optimal Θ using Equation (10). We can update the estimate at every iteration of gradient descent as

(12) $$\Theta_{m}=\Theta_{m-1}-\beta \nabla_{\Theta }L_{\Theta },$$

where β denotes the learning rate for the gradient descent.

In practice, we can also compute the gradient using auto-differentiation. In our experiments, we used Adam optimizer in PyTorch [65,66] to minimize the loss function in (8). A summary of the algorithm for learning the illumination patterns is also listed in Algorithm 2. Our code will be available at https://github.com/CSIPlab/learned-coded-diffraction (accessed on 12 December 2022).

## 4. Experiments

**Datasets.** We used MNIST digits, Fashion MNIST (F. MNIST), CIFAR10, SVHN, and CelebA datasets for training and testing in our experiments. We used 128 images from each of the datasets for training and another 1000 images for testing. To make the tiny-image datasets uniform, we reshaped all of them to 32×32 size with grayscale values. Images in CelebA dataset have 218×178 pixels. We first converted all the images to grayscale, cropped 178×178 region in the center, and resized them to 200×200.

**Measurements.** We used the amplitude of the 2D Fourier transform of the images modulated with *T* illumination patterns as the measurements. Unless otherwise mentioned, we used noiseless measurements. We report results for measurements with Gaussian and Poisson noise in Section 4.7.

**Computing platform.** We performed all the experiments using a computer equipped with Intel Core i7-8700 CPU and NVIDIA TITAN Xp GPU. We learned the illumination patterns using a PyTorch implementation, but we also implemented our algorithm in Matlab to provide a fair runtime comparison with existing phase retrieval methods.

### 4.1. Setup and Hyper-Parameter Search

The hyper-parameters include the number of iterations (*K*), step size α, and the number of training samples *N*. We set the default value of K=50, but we show in supplementary material that *K* can be adjusted as a trade-off between better reconstruction quality and shorter runtime. We tested all methods for T={2,3,4,8} to evaluate cases where signal recovery is hard, moderate, and easy. Through grid search, we found that it provides the best results over all datasets when α=4/T. We also studied the effect of the number of training images and found that illumination patterns learned on 32 randomly selected images provide good recovery over the entire dataset. The test accuracy improves slightly as we increase the number of training samples. To be safe, we used 128 training images in all our experiments. Unless otherwise mentioned, the images are constrained to be in [0,1] range for our experiments.

### 4.2. Comparison of Random and Learned Patterns

To demonstrate the advantages of our learned illumination patterns, we compared the performance of the learned and random illumination patterns on five different datasets. We learned a set of T={2,3,4,8} illumination patterns on 128 training images from a dataset and tested them on 1000 test images from the same dataset. For random patterns, we drew *T* independent patterns from uniform (0,1) distribution and tested their performance on the same 1000 samples that we used for the learned case. Unless otherwise mentioned, we repeated this process 30 times and chose the best result to compare with the results for the learned illumination patterns. The average peak signal-to-noise ratio (PSNR) over all 1000 test image reconstructions was presented in Table 1, which shows that the learned illumination patterns perform significantly better than the random patterns for all values of *T*. In addition to that, we observed a transition in the performance for T=3, where random patterns provided poor quality reconstructions and learned patterns provided reasonably high quality reconstructions. Furthermore, the learned patterns provided very high quality reconstructions for T≥4.

**Figure 2:**
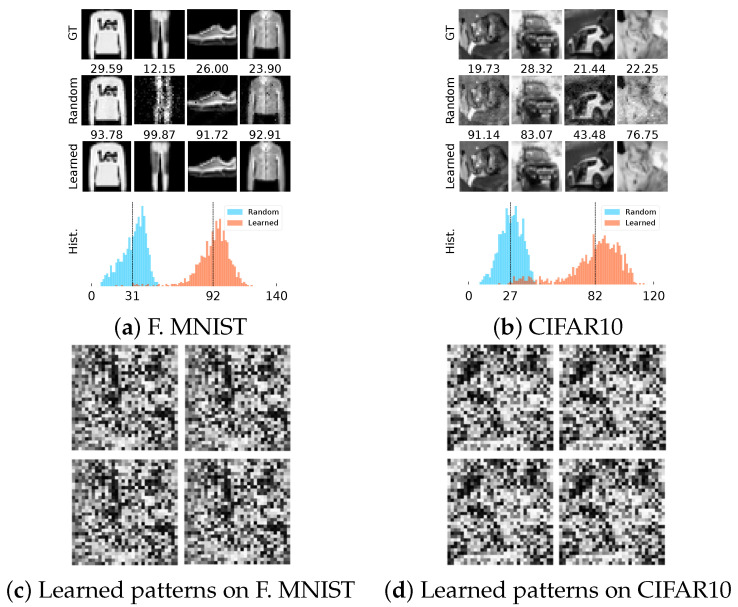
Reconstructed images using random and learned illumination patterns (T=4), along with ground truth (GT) in (**a**,**b**) and corresponding learned illumination patterns (**c**,**d**). PSNR is shown on top of every reconstruction. Below each dataset, we show the histograms of the PSNRs of all images with random patterns (shown in blue) and learned patterns (shown in orange). The dashed vertical line indicates the mean of all PSNRs.

To highlight this effect, we show a small set of reconstructed images and histograms of PSNRs of some reconstructed images from the learned and random illumination patterns in Figure 2 for T=4 patterns. The result suggests that the learned illumination patterns demonstrate consistently better performance compared to random illumination patterns. We demonstrate the corresponding learned illumination patterns in Figure 2. Visually, illumination patterns learned for the same dataset look similar, and patterns learned on different datasets look different.

### 4.3. Comparision with Existing Methods

We show a comparison with different existing methods using different datasets. Existing methods can be divided into four broad categories:

1. **AltMin methods:** Hybrid input output (HIO) [1] and Gerchberg–Saxton (GS) [25].
2. **Non-convex, gradient-based methods:** Wirtinger Flow [31] and Amplitude Flow [67].
3. **Convex method:** PhaseMax [35].
4. **Deep model-based method:** Deep S${}^{3}$PR [43].

We compare the performance of our method with these methods in terms of reconstruction quality and computation time. For algorithms in [1,25,30,31,35], we used the PhasePack [27] package. In our comparison, we used four illumination patterns and restricted all the illumination patterns in the range of [0,1]. For all the PhasePack algorithms, we used the default spectral initialization. We observed that different algorithms have different computational complexity in each iteration. Thus, a comparison in terms of the number of maximum iterations in all algorithms is not fair. To overcome this issue, we set the error tolerance ($\mathrm{tol}=10^{-6}$) and customized the maximum number of iterations in each algorithm to have comparable computations or performance. Specifically, we set the maximum iterations to be 100 for HIO and GS, and 2000 for Wirtinger Flow, Amplitude Flow, and PhaseMax. For our proposed method, we wanted to keep the number of iterations low (20, 50, 100). To make our runtime comparable with the PhasePack algorithms, we implemented our original Python code in Matlab.

For deep generative models, we used a modified version of the publicly available code for [43]. The code only provided pretrained DCGAN models for MNIST and F. MNIST; therefore, we trained our DCGAN models on the other datasets. This method is noticeably time-consuming because it optimizes over the latent vector for the deep model and uses 2000 iterations for each image where each iteration requires a forward and backward pass through the deep model. The patterns drawn from the uniform (0,1) range did not provide us good reconstruction with the Deep Model; therefore, we tested this method using random patterns drawn uniformly from the [−1,1] range and learned patterns that we manually scaled to [−1,1]. The reconstruction results for the Deep Model also directly depend on the quality of the trained generative models. In our experiments, we were not able to generate images with PSNR higher than 30dB using the generative models.

We tested all the methods using random illumination patterns and the learned illumination patterns using K=50 in our method. For the case of random illumination, we selected the best PSNR from five independent trials and report the average computation time for each experiment. In all the cases, we tuned the parameters that provide best results.

The reconstruction PSNR (in dB) and runtime (in seconds) per image are reported in Table 2 and Table 3, respectively. We observed that our proposed method with learned patterns performed significantly better than all other algorithms in terms of both reconstruction quality and runtime. We also observed that if we increase the number of iterations for other methods, their reconstruction quality improves beyond the numbers reported in Table 2, but this happens at the expense of much longer computation time.

### 4.4. Generalization on Different Algorithms

An interesting attribute of our learned patterns is that they can be used with different algorithms. Although we learned our illumination patterns using AltMin approach, it performs well for other algorithms. We observe in Table 2 that our learned patterns provide better results compared to Random patterns with almost all the phase retrieval algorithms for all the datasets, even though the patterns were not optimized for those algorithms. These results demonstrate the robust performance of our learned illumination patterns.

### 4.5. Generalization on Different Datasets

To explore the generalizability of our learned illumination patterns, we used patterns learned on one dataset to recover images from another. The results are shown in Table 4. As we can see in the table, the diagonal numbers are generally the best, and off-diagonal numbers are generally better than the ones with random illumination patterns.

### 4.6. Effect of Number of Iterations/Layers (K)

**Figure 3:**
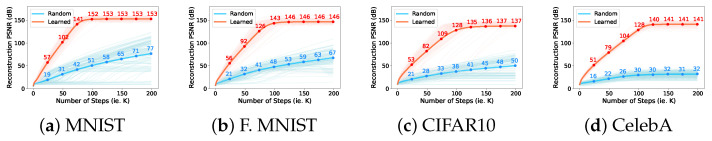
Comparison of the reconstruction quality with random (in blue) and learned (in red) illumination patterns for different values of K=1,…,200. We plot the average PSNR in a bright color and the PSNR of randomly selected 100 samples in light shadows.

Figure 3 shows the performance of the learned and random illumination patterns as we increased *K* to 200 at test time using the patterns learned for K=50. The number of illumination patterns is T=4. Random illumination patterns were selected as the best out of 30 trials. The learned illumination patterns were trained on 128 training images and number of iterations K=50 during training. We observed that with the learned patterns the image reconstruction process converges faster and is more stable (smaller variance) compared to the case with random patterns. The red curve in Figure 3 has a steeper slope and narrower shades. Besides the default setting for K=50, we also learn the illumination patterns for different values of *K*.

**Figure 4:**
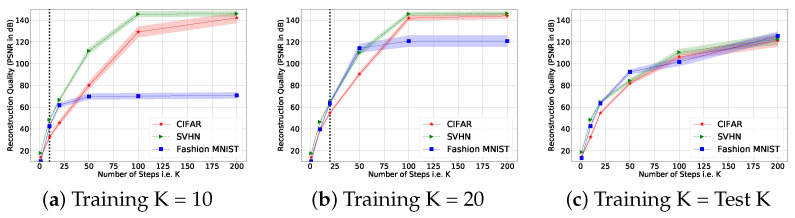
Reconstruction quality vs. number of iterations (layers) at test time (i.e., *K* is different for training and testing with T=4). We show an error bar of ±0.25σ for each dataset. In (**a**,**b**), we fixed *K* (*K* = 10, 20) and tested using different *K*. In (**c**), we trained and tested using the same number of layers.

Figure 4 shows that we can recover images in a small number of iterations if we use learned illumination patterns. We also observe that we can perform better if we use more iterations in testing than in training. We chose K=50 for most of the experiments as a trade-off between computational cost and reconstruction performance.

### 4.7. Noise Response

**Figure 5:**
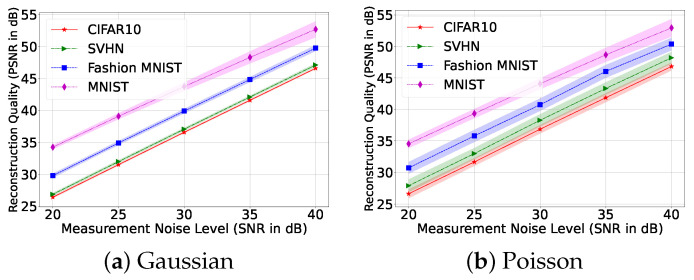
Reconstruction quality vs. noise level of the measurements for different datasets (T=4). Here, we show a shaded error bar of ±0.25σ for each dataset.

To investigate the robustness of our method to noise, we trained our illumination patterns on noiseless measurements obtained from the training datasets. We then added Gaussian and Poisson noise at different levels to the measurements from the test datasets. Poisson noise or shot noise is the most common in the imaging systems, which we add following the approach in [20,68]. Let us denote the $i^{th}$ element of measurement vector corresponding to $t^{th}$ illumination pattern, $y_{t}$ as

(13) $$y_{t}\left(i\right)=\left|z_{t}\left(i\right)\right|+\eta_{t}\left(i\right),\;\;\;\mathrm{for}\;i=1,2,\ldots ,m,$$

where $\eta_{t}\left(i\right)∼N(0,\lambda |z_{t}\left(i\right)\left|\right)$, and $z_{t}=F\left(d_{t}⊙x\right)$. We varied λ to generate noise at different signal-to-noise ratio (SNR) levels. Poisson noise affects larger values in measurements with higher strength than the smaller values. Since the sensors can measure only positive measurements, we kept the measurements positive by applying the ReLU function after noise addition. We expected the reconstruction to be affected by noise as we did not use any denoiser. We observe the effect of noise in Figure 5 with illumination patterns learned under a noiseless setup. Even though noise affects the reconstructions, we can obtain reasonable reconstruction up to a certain level of noise. The relationship between noise level and reconstruction performance also indicates that our phase retrieval system is quite stable.

We ran another set of experiments where we learned a different set of illumination patterns at different noise levels by introducing measurement noise during training. In Table 5, we report results for the MNIST and CIFAR10 datasets at different levels of Poisson noise introduced during training and testing. We show the performance of some comparable approaches to our learned patterns and random patterns. For random patterns, we reported the results for the best out of five runs. We can observe that even under the presence of high noise (0–20 dB), the learned illumination patterns using our approach performed reasonably well. We observed a performance boost with our learned patterns for 5 dB or higher SNR.

### 4.8. Mismatch in Training and Test Images

**Figure 6:**
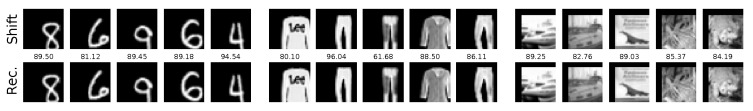
Test results on images shifted to bottom right by 5 pixels; from **left** to **right**: MNIST, F. MNIST, and CIFAR10.

**Figure 7:**
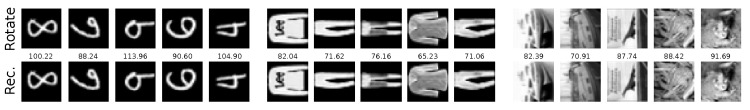
Test results on images rotated by 90${}^{\circ }$; from **left** to **right**: MNIST, F. MNIST, and CIFAR10.

In our final experiment, we tested illumination patterns trained on upright images to recover shifted and rotated images. Our results in Figure 6 and Figure 7 show that the learned patterns reliably recovered images regardless of the position or orientation. This is not surprising because we do not learn to represent images or solve the phase retrieval problem using the training data; instead, we only learned the illumination patterns using a predefined AltMin-based recovery algorithm. In contrast, data-driven methods that learn to solve the inverse problem may suffer if the distribution of test images differs significantly from the training images.

## 5. Conclusions

We presented a framework to learn the illumination patterns for coded diffraction imaging by formulating an iterative phase retrieval algorithm as a fixed unrolled network. We learned the illumination patterns using a small number of training images via backpropagation. Our results demonstrate that the learned patterns provide near-perfect reconstruction, whereas random patterns fail. The number of iterations in our algorithm provides a clear trade-off between reconstruction accuracy and runtime. In addition, the learning process of our illumination patterns is highly data efficient and requires only a small number of training samples. The learned patterns generalize to different datasets and algorithms that were not used during training.

## Figures and Tables

**Table 1:** PSNR (mean ± std) for random and learned illumination patterns.

Dataset	2 Patterns		3 Patterns		4 Patterns		8 Patterns	
	Random	Learned	Random	Learned	Random	Learned	Random	Learned
MNIST	14 ± 6	28 ± 9	20 ± 11	75 ± 19	32 ± 14	102 ± 10	61 ± 19	113 ± 11
F. MNIST	17 ± 4	26 ± 6	20 ± 6	49 ± 15	33 ± 9	94 ± 13	67 ± 14	111 ± 12
CIFAR10	15 ± 3	26 ± 4	20 ± 3	34 ± 10	30 ± 8	86 ± 18	64 ± 15	108 ± 18
SVHN	17 ± 3	28 ± 6	24 ± 4	45 ± 15	35 ± 7	93 ± 21	73 ± 15	118 ± 21
CelebA	13 ± 2	19 ± 3	14 ± 4	28 ± 2	23 ± 5	81 ± 4	43 ± 8	98 ± 15

**Table 2:** Reconstruction PSNR (mean ± std) of different algorithms using random patterns and our learned patterns (T=4).

	MNIST		F. MNIST		CIFAR10		SVHN		CelebA	
	Random	Learned	Random	Learned	Random	Learned	Random	Learned	Random	Learned
HIO [1]	16 ± 9	37 ± 19	32 ± 14	61 ± 24	49 ± 20	99 ± 25	60 ± 22	114 ± 27	38 ± 5	102 ± 5
GS [25]	16 ± 9	37 ± 19	33 ± 15	61 ± 24	48 ± 20	99 ± 25	60 ± 22	114 ± 27	38 ± 4	102 ± 5
WirtFlow [31]	22 ± 16	48 ± 25	33 ± 14	51 ± 19	41 ± 10	57 ± 10	41 ± 10	58 ± 10	20 ± 2	39 ± 3
AmpFlow [30]	42 ± 32	74 ± 48	64 ± 38	109 ± 43	86 ± 37	138 ± 25	97 ± 33	144 ± 21	42 ± 8	138 ± 11
PhaseMax [35]	14 ± 4	24 ± 8	21 ± 4	45 ± 20	26 ± 4	97 ± 41	32 ± 5	115 ± 33	32 ± 2	148 ± 2
Ours − K = 20	17 ± 6	49 ± 8	20 ± 6	49 ± 8	21 ± 6	49 ± 9	26 ± 5	55 ± 11	16 ± 4	46 ± 3
Ours − K = 50	32 ± 14	102 ± 10	33 ± 9	94 ± 13	30 ± 8	86 ± 18	35 ± 7	93 ± 21	23 ± 5	81 ± 4
Ours − K = 100	51 ± 19	186 ± 15	49 ± 11	162 ± 22	40 ± 10	139 ± 30	45 ± 10	149 ± 35	33 ± 4	132 ± 7
Deep Model [43]	31 ± 2	32 ± 3	22 ± 4	22 ± 4	28 ± 3	25 ± 3	26 ± 3	28 ± 4	22 ± 3	23 ± 2

**Table 3:** Average runtime (sec) per image of different algorithms corresponding to the performance reported in Table 2. The reported runtime corresponds to the time required for convergence of each algorithm. ‡ Image size for CelebA generator is 64 × 64.

		HIO [1]	GS [25]	Wirt- Flow [31]	Amp- Flow [30]	Phase- Max [35]	Deep Model [43]	Ours K = 20	Ours K = 50	Ours K = 100
Max iterations		100	100	2000	2000	2000	2000	20	50	100
Image size	32×32	0.473	0.461	0.459	0.080	0.563	8.422	0.008	0.011	0.017
	200×200	7.353	7.269	10.90	2.377	10.84	10.55 ‡	0.061	0.124	0.238

**Table 4:** Reconstruction PSNR (mean ± std) of illumination patterns learned and tested on different datasets for K=50. Every column corresponds to patterns learned on a fixed dataset and tested on all. The random column reports the performance of random illumination patterns.

Test\Train	4 Illumination Patterns					8 Illumination Patterns				
	MNIST	F. MNIST	CIFAR10	SVHN	Random	MNIST	F. MNIST	CIFAR10	SVHN	Random
MNIST	102 ± 10	66 ± 16	34 ± 15	48 ± 15	32 ± 14	113 ± 11	84 ± 13	56 ± 20	74 ± 19	61 ± 19
F. MNIST	84 ± 24	94 ± 13	50 ± 20	64 ± 19	33 ± 9	94 ± 23	111 ± 12	89 ± 20	108 ± 21	67 ± 14
CIFAR10	79 ± 27	87 ± 13	86 ± 18	96 ± 17	30 ± 8	84 ± 18	88 ± 17	108 ± 18	113 ± 17	64 ± 15
SVHN	56 ± 28	78 ± 16	72 ± 21	93 ± 21	35 ± 7	76 ± 19	95 ± 12	91 ± 24	118 ± 21	73 ± 15

**Table 5:** Reconstruction PSNR (mean ± std) of different algorithms using random patterns (best out of 5 trials) and our learned patterns (T=4) at different Poisson noise levels for the MNIST and CIFAR10 datasets.

Noise SNR	HIO [1]		GS [25]		WirtFlow [31]		PhaseMax [35]		Ours − K = 50	
	Random	Learned	Random	Learned	Random	Learned	Random	Learned	Random	Learned
MNIST										
0	23 ± 13	25 ± 15	16 ± 9	25 ± 15	20 ± 16	25 ± 15	16 ± 5	18 ± 6	28 ± 16	24 ± 3
5	17 ± 10	19 ± 12	19 ± 11	18 ± 12	23 ± 19	23 ± 18	13 ± 3	16 ± 6	21 ± 13	28 ± 5
10	22 ± 12	18 ± 10	22 ± 13	18 ± 10	27 ± 20	25 ± 16	15 ± 5	16 ± 5	28 ± 12	31 ± 5
20	18 ± 11	23 ± 16	20 ± 11	22 ± 16	29 ± 20	28 ± 22	17 ± 5	17 ± 6	16 ± 11	48 ± 13
30	22 ± 11	10 ± 3	21 ± 11	10 ± 3	30 ± 19	14 ± 9	17 ± 5	11 ± 2	22 ± 13	65 ± 21
40	20 ± 11	11 ± 4	17 ± 8	11 ± 4	31 ± 19	24 ± 16	16 ± 4	11 ± 2	27 ± 13	61 ± 17
CIFAR10										
0	28 ± 26	18 ± 16	27 ± 27	17 ± 15	23 ± 20	16 ± 14	17 ± 12	23 ± 22	29 ± 6	26 ± 9
5	28 ± 28	20 ± 18	26 ± 25	19 ± 18	23 ± 19	18 ± 16	16 ± 10	23 ± 22	28 ± 7	30 ± 12
10	27 ± 25	31 ± 31	32 ± 30	33 ± 32	23 ± 18	23 ± 22	16 ± 11	29 ± 32	29 ± 7	38 ± 10
20	28 ± 25	41 ± 42	27 ± 26	45 ± 42	23 ± 20	31 ± 28	16 ± 11	50 ± 55	28 ± 5	51 ± 10
30	28 ± 26	47 ± 43	27 ± 26	47 ± 43	23 ± 19	30 ± 28	17 ± 11	48 ± 52	30 ± 8	68 ± 12
40	29 ± 27	51 ± 44	29 ± 26	51 ± 44	24 ± 20	33 ± 30	18 ± 12	58 ± 60	31 ± 7	71 ± 9

## Data Availability

We used MNIST digits, Fashion MNIST, CIFAR10, SVHN, and CelebA datasets for training and testing in our experiments. All of these 5 datasets are publicly available (MNIST digits: http://yann.lecun.com/exdb/mnist/ accessed on 15 November 2022, Fashion MNIST: https://github.com/zalandoresearch/fashion-mnist accessed on 15 November 2022, CIFAR10: https://www.cs.toronto.edu/~kriz/cifar.html accessed on 15 November 2022, SVHN: http://ufldl.stanford.edu/housenumbers/ accessed on 15 November 2022, and CelebA: https://mmlab.ie.cuhk.edu.hk/projects/CelebA.html accessed on 15 November 2022).
